# Lytic transglycosylase MltG cleaves in nascent peptidoglycan and produces short glycan strands

**DOI:** 10.1016/j.tcsw.2021.100053

**Published:** 2021-05-01

**Authors:** Jad Sassine, Manuel Pazos, Eefjan Breukink, Waldemar Vollmer

**Affiliations:** a Centre for Bacterial Cell Biology, Biosciences Institute, Faculty of Medical Sciences, Newcastle University, Newcastle upon Tyne, UK; b Membrane Biochemistry and Biophysics, Bijvoet Centre of Biomolecular Research, Department of Chemistry, Faculty of Science, Utrecht University, Utrecht, Netherlands

**Keywords:** PBP, penicillin-binding protein, PG, peptidoglycan, TPase, transpeptidase, GTase, glycosyltransferase, LT, lytic transglycosylase, Peptidoglycan, Lytic transglycosylase, Penicillin-binding protein

## Abstract

Bacteria encase their cytoplasmic membrane with peptidoglycan (PG) to maintain the shape of the cell and protect it from bursting. The enlargement of the PG layer is facilitated by the coordinated activities of PG synthesising and -cleaving enzymes. In *Escherichia coli*, the cytoplasmic membrane-bound lytic transglycosylase MltG associates with PG synthases and was suggested to terminate the polymerisation of PG glycan strands. Using pull-down and surface plasmon resonance, we detected interactions between MltG from *Bacillus subtilis* and two PG synthases; the class A PBP1 and the class B PBP2B. Using *in vitro* PG synthesis assays with radio-labelled or fluorophore-labelled *B. subtilis*-type and/or *E. coli*-type lipid II, we showed that both, *Bs*MltG and *Ec*MltG, are lytic tranglycosylases and that their activity is higher during ongoing glycan strand polymerisation. MltG competed with the transpeptidase activity of class A PBPs, but had no effect on their glycosyltransferase activity, and produced glycan strands with a length of 7 disaccharide units from cleavage in the nascent strands. We hypothesize that MltG cleaves the nascent strands to produce short glycan strands that are used in the cell for a yet unknown process.

## Introduction

Most bacteria are engulfed by peptidoglycan (PG), a mesh-like molecule that maintains the shape of the cell and protects it from bursting due to the turgor (Vollmer et al., 2008). Growing cells continuously synthesise, modify and cleave PG to maintain cell integrity (Vollmer et al., 2008). PG is synthesised from the precursor lipid II. Glycosyltransferases (GTases) polymerize glycan strands and DD-transpeptidases (TPases) form peptide crosslinks (Barrett et al., 2007, Egan et al., 2020, Lovering et al., 2012, Van Heijenoort, 2001). PG synthases comprise bifunctional class A penicillin-binding proteins (PBPs) with GTase and TPase activity, SEDS and Mtg proteins with GTase activity, and monofunctional class B PBPs with TPase activity (Egan et al., 2015, Meeske et al., 2016, Taguchi et al., 2019).

In *Bacillus subtilis, Bs*PBP1 is the most abundant class A PBP with roles in PG synthesis during length growth and cell division (Claessen et al., 2008, Pedersen et al., 1999). *Escherichia coli* has two semi-redundant class A PBPs, *Ec*PBP1A and *Ec*PBP1B, with preferential roles in elongation and cell division, respectively (Banzhaf et al., 2012, Bertsche et al., 2006, Yousif et al., 1985). *Ec*PBP1A and *Ec*PBP1B are activated by the outer membrane-anchored lipoproteins LpoA and LpoB, respectively (Paradis-Bleau et al., 2010, Typas et al., 2010). The SEDS proteins *Bs*FtsW and *Ec*FtsW interact with their cognate class B PBP, *Bs*PBP2B and *Ec*PBP3, respectively, and synthesise PG in the test tube (Fraipont et al., 2011, Rohs et al., 2018, Sjodt et al., 2018, Taguchi et al., 2019).

In *B. subtilis* and *E. coli*, glycan strands terminate with 1,6-anhydroMurNAc residues that harbours an intramolecular ring from C1 to C6 (Atrih et al., 1999, Glauner et al., 1988, Höltje et al., 1975). AnhydroMurNAc sugars are synthesised by lytic transglycosylases (LTs) that catalyse the non-hydrolytic cleavage of the glycosidic bond between the N-acetylmuramic acid and N-acetylglucosamine (Höltje et al., 1975). *B. subtilis* has five known or predicted LTs, YomI, SleB, CwlQ, YuiC and SleC, of which some may play roles in the lysis of the spore cortex for germination (Kumazawa et al., 2007, Quay et al., 2015, Sudiarta et al., 2010, Smith et al., 2000). *E. coli* has nine LTs, of which MltA, MltB, MltC, MltD, MltE, MltF and RlpA are lipoproteins anchored to the outer membrane (Jorgenson et al., 2014, van Heijenoort, 2011), Slt is a periplasmic enzyme (Höltje et al., 1975), and *Ec*MltG an inner membrane protein (Yunck et al., 2016).

Bioinformatics analysis showed that *Ec*MltG and its defining YceG domain are widely distributed in the bacterial domain (Yunck et al., 2016). *Ec*MltG is not essential and cells lacking *Ec*MltG have no growth defect but the overexpression of *Ec*MltG in cells lacking the synthase *Ec*PBP1B resulted in loss of rod shape and cell death (Yunck et al., 2016). Purified *Ec*MltG has a weak activity against PG causing the release of glycan strands with anhydroMurNAc ends, and *Ec*MltG associates with *Ec*PBP1B and RodA but not with *Ec*PBP1A, GTase inactive *Ec*PBP1B E233Q or RodA D262A in bacterial two-hybrid experiments (Bohrhunter et al., 2020, Yunck et al., 2016), hence, *Ec*MltG has been hypothesized to act as a terminase of glycan strand synthesis. In *Streptococcus pneumoniae, Sp*MltG has an additional cytoplasmic domain of unknown function and the loss of *Sp*MltG resulted in a growth defect and a more spherical cell shape (Tsui et al., 2016).

*B. subtilis* has two MltG homologues with a predicted YceG-like domain, the forespore germination-specific SleB (Moriyama et al., 1999), and YrrL. Here, we focused on YrrL which has 32% amino acid sequence identity and 50% similarity with *Ec*MltG extending over 356 amino acid residues (Fig. S1). We renamed YrrL to *Bs*MltG. We show that purified *Bs*MltG interacts with several PBPs and that it has lytic transglycosylase activity on nascent glycan strands, but is inactive against crosslinked PG, producing short glycan strands with a length of 7 disaccharide residues.

## Methods

### Media and general methods

Bacterial strains and plasmids used in this work are listed in Tables S1 and S2, respectively, and primers are listed in Table S3. *E. coli* cells were cultivated in Luria Britani (LB). Fresh cultures were inoculated with overnight culture and grown at 30 or 37˚C with continuous shaking. For solid media, 1% agar (Bacteriological agar no. 1, Oxoid) was added in addition to the appropriate antibiotic concentration (50 µg/ml Kanamycin). Competent *E. coli* cells were produced according to Hanahan et al. (1991), and heat shock transformations were used for DH5α as described by Bergmans et al. (1981). DNA was purified using DNA purification kit (Quiagen) as per manufacturer's instructions.

### Cloning of strains and plasmids

Plasmids were generated using a ligase-free cloning method, which requires 4 sets of primers and 2 DNA templates: plasmid DNA and genomic DNA. pET-28a(+) plasmid was amplified using JS128-JS129 primers, and inserts were amplified using the oligonucleotides in Table S3. Plasmid construction was performed as described in Richardson et al. (2016). For site directed mutagenesis, a PCR reaction was performed using NEB Q5 high fidelity polymerase, plasmid construct as a template, and the corresponding primers from Table S3. Plasmids were purified using Mini-prep kit (Quiagen) as per manufacturer’s instruction.

### Protein purification

*BsMltG, BsMltG E242A, BsPBP2B, EcMltG and EcMltG E218Q*. BL21 (DE3) cells with the corresponding plasmids were grown in LB medium at 37˚C to OD600 0.5. Gene expression was induced with 1 mM IPTG for 3 h at 30˚C. Cells were pelleted by centrifugation (6371 × *g* / 4˚C/ 15 min) and resuspended in 40 ml buffer I (25 mM Tris/HCl, 1 M NaCl, pH 7.5) supplemented with protease inhibitor cocktail (PIC), phenylmethylsulfonyl fluoride (PMSF) and DNase (≈ 1 mg). Cells were sonicated for 3 × 20 s at 5, 16, 22, 33, 44 and 60 W. Membrane proteins were pelleted by ultracentrifuge at 133,907 × *g* at 4˚C, for 1 h, and the soluble fraction was discarded. The pellet was resuspended in running buffer (25 mM Tris/HCl, 1 M NaCl, 20 mM imidazole, 2% Triton X-100, pH 7.5). Solubilised proteins were mixed gently with equilibrated Ni-NTA beads for 24 h at 4˚C. The mixture was applied to a gravity column and bound proteins were eluted in elution buffer (25 mM Tris/HCl, 400 mM imidazole, 1 M NaCl, 0.2% Triton X-100, pH 7.5). Restriction grade thrombin was added to eluted proteins, and samples were dialysed against 2 × 2 l of dialysis buffer I (25 mM Tris/HCl, 500 mM NaCl, pH 6.5) and 2 × 2 l of dialysis buffer II (25 mM Tris/HCl, 100 mM NaCl, pH 6.5) for overnight at 4˚C. Ion exchange chromatography was performed using an Äkta Prime FPLC with a HiTrap SP HP column. Dialysed protein was injected onto the column equilibrated with buffer I (25 mM Tris/HCl, 100 mM NaCl, 0.2% Triton X-100, pH 6.5). Bound protein was eluted in a 50 ml gradient to buffer II (25 mM Tris/HCl, 1 M NaCl, 0.2% Triton X-100, pH 7.5).

*PBP1 and PBP1 (S390A)*. The purification protocol was adopted from (Rismondo et al., 2016) and modified. BL21 (DE3) cells with corresponding plasmids were grown in 5 l of LB medium with 50 μg/ml kanamycin and 20 ml/l auto-induction medium (250 mg/l glycerol, 100 g/L α-lactose and 25 g/L glucose) for 18 h at 30˚C. Cells were harvested, and sonicated. Cell membranes were pelleted by ultracentrifuge at 133907 × *g* then resuspended in resuspension buffer (50 mM Hepes/NaOH, 500 mM NaCl, 3 mM MgCl_2_, 2% Triton X-100, 15% glycerol, 10 mM β-mercaptoethanol, pH 7.5). Membrane extracts were ultracentrifuged at 100000 × *g* and the supernatant was supplied with 20 mM imidazole. The first purification step was performed using a 5 ml HisTrap HP column attached to an ÄKTA Prime plus FPLC. Proteins were injected using running buffer (50 mM Hepes/NaOH, 500 mM NaCl, 3 mM MgCl_2_, 0.2% reduced Triton X-100, 15% glycerol, 20 mM imidazole, pH 7.5) and eluted with elution buffer (same as running buffer with 250 mM imidazole). Protein samples were mixed with restriction grade thrombin and dialysed overnight at 4˚C against 3 l dialysis buffer (25 mM Hepes/NaOH, 300 mM NaCl, 10% glycerol, 0.2% Triton X-100, pH 8.5). Next, ion exchange chromatography was performed using an Äkta Prime FPLC with a HiTrap SP HP column. Dialysed protein samples were injected into the column using buffer I (Same as the dialysis buffer with 0.2% Triton X-100) and eluted in a 200 ml gradient with buffer II (10 mM Hepes/NaOH, 1 M NaCl, 3 mM MgCl_2_, 0.2% Triton X-100, 12% glycerol, pH 7.5). The third purification step consisted of a size exclusion chromatography using a Superdex 75 column. Proteins were eluted with SEC buffer (10 mM Hepes/NaOH, 300 mM NaCl, 3 mM MgCl_2_, 0.2% Triton X-100, 12% glycerol, pH 7.5).

PBP1B, PBP1B S510A and LpoB were purified as described in (Born et al., 2006). PBP1A and LpoA were purified as described in (Born et al., 2006) and (Jean et al., 2014), respectively.

### In vitro pulldown assay

This method was adapted from (Egan et al., 2015). Proteins (1 μM) were mixed in 200 μl binding buffer (10 mM Hepes/NaOH, 10 mM MgCl_2_, 150 mM NaCl, 0.05% Triton X-100, pH 7.5). Samples were applied to 100 μl of washed and equilibrated Ni-NTA superflow beads (Qiagen, The Netherlands) and incubated overnight at 4˚C with gentle mixing. The beads were then washed with wash buffer (10 mM Hepes/NaOH, 10 mM MgCl_2_, 500 mM NaCl, 50 mM imidazole, 0.05% Triton X-100, pH 7.5) and boiled in SDS–PAGE loading buffer. Beads were pelleted by centrifugation and samples analysed by SDS–PAGE. Gels were stained with Coomassie brilliant blue (Roth, Germany).

### Surface Plasmon Resonance (SPR) assay

This method was adapted from (Vollmer et al., 1999). Binding assays were performed at 25˚C in running buffer (10 mM Tris/Maleate, 150 mM NaCl, 0.05% Triton X-100, pH 7.5). Proteins to be injected (analyte) were dialysed into 2 × 1 l of dialysis buffer (10 mM Tris/Maleate, 150 mM NaCl, pH 7.5) then centrifuged using a Beckman TLA120.2 rotor (90000 *rpm*, 30 min, 4˚C) to remove aggregates. The concentration of the protein was measured and the analytes were diluted in running buffer to 6 concentration ranges from 0 to 500 nM. It was important to make sure the Triton X-100 level in the analyte was as close to the running buffer as possible. SigmaPlot software (windows version 13.0) was used for kinetic calculations. Several repeats (at least 3) were required across a range of analyte concentrations. The K_D_ (nM) of ligand binding was based on the assumption of a one site saturation with the use of the equation y = Bmax × x(K_D_ + x) where y is the response (RU) for an analyte concentration in (nM), and Bmax is the maximum response recorded (RU).

### In vitro glycosyltransferase activity assay

For assay using radio-labelled *B. subtilis* or *E. coli* lipid II, experiments were performed as published (Egan and Vollmer, 2016). For assay using ATTO-550 lipid II, experiments were performed as published in (Barrett et al., 2007, van’t Veer et al., 2016) with modifications according to (Egan et al., 2018). Glycan strands were separated by Tris-Tricine SDS-PAGE.

### In vitro peptidoglycan synthesis assay

This method was performed as in (Cleverley et al., 2016) for *Bs*PBP1, and as in (Bertsche et al., 2005) for *Ec*PBP1B and *Ec*PBP1A. For HPLC analysis, a linear gradient was used at 55˚C for 90 min, from 100% solvent A (50 mM sodium phosphate pH 4.31 + 0.0002% NaN3) to 100% solvent B (75 mM sodium phosphate, 15% methanol, pH 4.75) for *Bs*PBP1 and *Ec*PBP1B samples, and solvent B (75 mM sodium phosphate, 30% methanol, pH 4.75) for *Ec*PBP1A samples.

### Cell wall purification and muropeptide analysis

This method was adapted from (Atrih et al., 1999) and modified as per (Bisicchia et al., 2011).

## Results

### BsMltG interacts with BsPBP1 and BsPBP2B

**Fig. 1:**
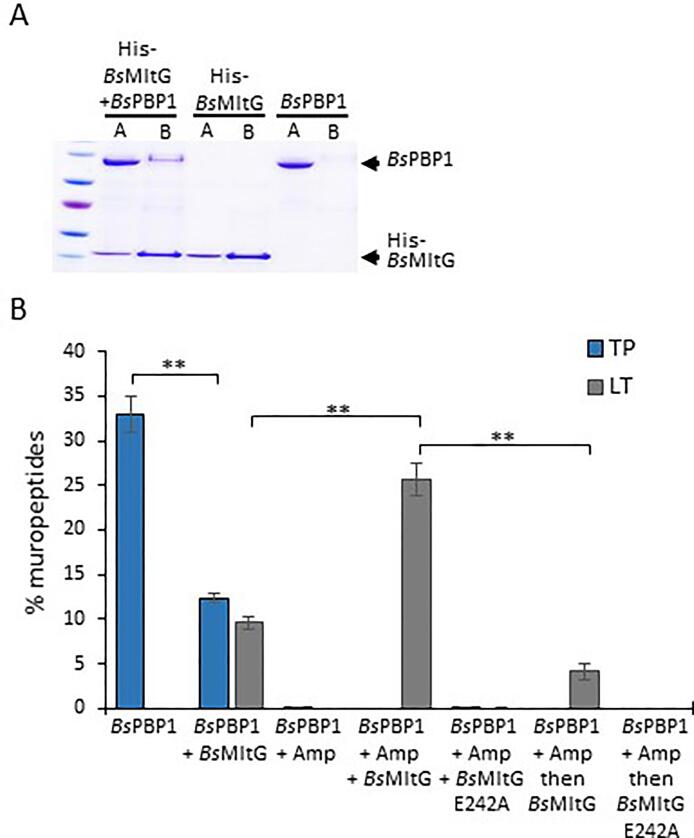
*Bs*MltG interacts with *Bs*PBP1 and is active during ongoing glycan strand polymerisation. (A) Pull-down assays performed to test if *Bs*MltG interacts *Bs*PBP1. The Coomassie-stained SDS-PAGE analysis shows His-*Bs*MltG and *Bs*PBP1 in the applied and bound fractions suggesting that His-*Bs*MltG pulled down *Bs*PBP1. A, Applied; B, bound fractions. (B) Level of crosslinked and anhydro-N-acetylmuramic acid containing muropeptides generated in reactions with *Bs*PBP1 or *Bs*MltG versions, or combinations, in assays with the *B. subtilis*-type lipid II. The PG product was digested with cellosyl and the resulting muropeptides were reduced and separated by HPLC. Representative HPLC chromatograms are shown in Fig. S4. Values are the mean ± standard deviation of three independent experiments. *Bs*MltG has lytic transglycosylase activity in the presence of *Bs*PBP1. *Bs*MltG reduces the TPase activity of *Bs*PBP1. Blue columns: TPase products (TP); Grey columns: LT products (LT); MltG E242A, catalytically inactive version; Amp; Ampicillin. **, P < 0.01 (T-test). (For interpretation of the references to colour in this figure legend, the reader is referred to the web version of this article.)

*Ec*MltG presumably associates with *Ec*PBP1B based on bacterial two-hybrid experiments (Yunck et al., 2016). To test if *Bs*MltG interacts with a class A PBP, we purified hexahistidine tagged *Bs*MltG (His-*Bs*MltG) and tested its possible interaction with *Bs*PBP1 in a pull-down experiment. SDS-PAGE analysis shows that His-*Bs*MltG bound to Ni-NTA, and *Bs*PBP1 was not retained by the beads in the absence of His-*Bs*MltG (Fig. 1A). However, *Bs*PBP1 was present in the bound fraction when applied together with His-*Bs*MltG suggesting the two proteins interact. The interaction between *Bs*MltG and *Bs*PBP1 was also tested by surface plasmon resonance (SPR), but due to the high level of unspecific binding of proteins to the chip surface, we were not able to confirm the interaction by SPR.

We also tested if His-*Bs*MltG interacts with the monofunctional cell division-specific *Bs*PBP2B in pull-down experiment. SDS-PAGE analysis shows that His-*Bs*MltG bound to Ni-NTA and *Bs*PBP2B was not pulled down by the beads in the absence of His-*Bs*MltG (Fig. S2A). Interestingly, *Bs*PBP2B in the presence of His-*Bs*MltG appeared in both the pre-Ni-NTA sample (applied) and bound fractions indicating that His-*Bs*MltG pulled down *Bs*PBP2B and suggesting that *Bs*PBP2B interacts with His-*Bs*MltG.

Next, we used SPR to study the interaction between *Bs*MltG and *Bs*PBP2B and determine the dissociation constant. We first immobilized ampicillin (amp) on two traces of a sensor chip. On one trace, PBP2B was covalently bound to the immobilized ampicillin (via its TPase domain). The second trace received no *Bs*PBP2B and served as control. We next incubated both surfaces with β-lactamase to digest ampicillin molecules not bound to PBP2B. Upon the injection of *Bs*MltG, the surface with immobilized PBP2B showed a significant increase in response unit during association (from 0 to 300 s) compared to the control surface upon the injection of *Bs*MltG (Fig. S2B). The response almost reached equilibrium towards the end of the association (from 200 to 300 s). Subsequently, running buffer was injected after 300 s causing the dissociation of the analyte. These results indicate an interaction between *Bs*MltG and *Bs*PBP2B. The binding curve was generated by plotting the response in (RU) during equilibrium against analyte concentration (Fig. S2C). The K_D_ of the *Bs*MltG and *Bs*PBP2B interaction was 128 ± 28 nM, calculated based on a one site interaction model, from three independent experiments using Sigma Plot software. These results suggest that *Bs*MltG interacts with both class A and B PBPs during PG synthesis in *B. subtilis*.

### LT activity of BsMltG

*Ec*MltG showed weak LT activity against purified *Ec*PG, and the loss of *Ec*MltG in cells resulted in longer glycan strands (Yunck et al., 2016)*.* At first, we tested *Bs*MltG LT activity against *B. subtilis* PG, however, *Bs*MltG did not release muropeptides, suggesting that *Bs*MltG was not active against PG (Fig. S3). Next, assuming that *Bs*MltG processes glycan strands, and supported by the observed interaction of *Bs*MltG with *Bs*PBP1 and *Bs*PBP2B, we hypothesised that *Bs*MltG might be active during ongoing PG synthesis. Since SEDS proteins were shown to be active only in the presence of 20–30% DMSO, which is known to disrupt some protein–protein interactions (Egan et al., 2018), we characterised the activity of MltG in the presence of class A PBPs which are active in the absence of DMSO. Consequently, the activity of purified *Bs*MltG and *Bs*PBP1 were tested *in vitro* against the *B. subtilis* type lipid II (with amidated *meso*-Dap at position 3). A catalytically-inactive *Bs*MltG variant possessing an Ala residue instead of the putative catalytic Glu242 was used in control experiments. *Bs*PBP1 polymerised glycan strands and crosslinked peptide stems producing PG, which was digested by cellosyl and analysed by HPLC (Fig. S4). *Bs*PBP1 alone produced a PG with 32.9% of the peptides participating in crosslinks, whereas, *Bs*MltG alone had no activity against lipid II (Fig. 1B, S4). *Bs*PBP1 in the presence of *Bs*MltG produced a PG with significantly reduced level of peptides in crosslinks (12.3%), and we noticed an additional peak (9.6%) corresponding to GlcNAc-anhydroMurNAc-pentapeptide (PentaAnh), identified by LC-MS/MS with 991.4340 amu (neutral mass, theoretical: 991.4346), eluting at 64 min. The peak corresponding to PentaAnh was not present in samples with *Bs*PBP1 and *Bs*MltG E242A (Fig. S4), confirming that the E242 glutamate residue is essential for *Bs*MltG LT activity. These results suggest that *Bs*MltG has LT activity and reduces the TPase activity of *Bs*PBP1.

Previously, *Ec*MltG was shown to release only uncrosslinked anhydroMurNAc muropeptides from purified PG (Yunck et al., 2016) and it might therefore favour uncrosslinked glycan strands for activity. Consequently, we added ampicillin to samples to block the TPase activity of *Bs*PBP1 (Fig. 1B and S4). *Bs*MltG produced significantly more (25.7%) LT product in the presence of ampicillin-inhibited *Bs*PBP1, suggesting that *Bs*MltG shows enhanced activity against uncrosslinked, nascent PG strands. Next, to test if *Bs*MltG requires ongoing PG synthesis by *Bs*PBP1 for activity, glycan strands were first synthesised by *Bs*PBP1 in the presence of ampicillin followed by digest with *Bs*MltG (*Bs*PBP1 + Amp then *Bs*MltG sample, Fig. 1B, and S4). In this sample there was approximately 5 times fewer LT products present as in the sample in which *Bs*MltG was present during glycan strand synthesis, suggesting that *Bs*MltG is most active during ongoing glycan strand polymerisation.

To test if *Bs*MltG relies on both, ongoing PG synthesis and its interaction with *Bs*PBP1, for higher activity, its activity was tested against nascent PG strands polymerized from *B. subtilis* lipid II by *Ec*PBP1B (instead of *Bs*PBP1). *Bs*MltG was less active in the sample with *Ec*PBP1B compared to *Bs*PBP1, producing 4.5% PentaAnh (Fig. S5). Additionally, *Bs*MltG showed high activity against glycan strands produced by *Bs*PBP1 from *E. coli* type lipid II (with non-amidated *meso*-Dap), suggesting that the amidation of the *meso*-DAP has no effect on the activity of *Bs*MltG (Fig. S5).

To test if *Bs*MltG has an *endo*-LT activity, PG material synthesised by PBP1 in the presence of ampicillin and *Bs*MltG was split into two aliquots, one was digested with the muramidase cellosyl and the other not, followed by HPLC analysis. An *exo*-LT activity of *Bs*MltG should generate PentaAnh, which elutes in 1 peak at 64 min. However, PentaAnh was not present in the sample with *Bs*MltG that was not digested with cellosyl. The sample digested first with *Bs*MltG and then with cellosyl contained PentaAnh (from the MurNAcAnh termini of the glycan strands) and Penta (from within the glycan strands and the GlcNAc termini) at a ratio of 1/5.2 (Fig. S5), showing that *Bs*MltG was an *endo*-specific lytic transglycosylase that released glycan strands with an average length of 5–6 disaccharide units from nascent PG strands synthesized by PBP1.

### EcMltG reduces the TPase activity of EcPBP1A and EcPBP1B

**Fig. 2:**
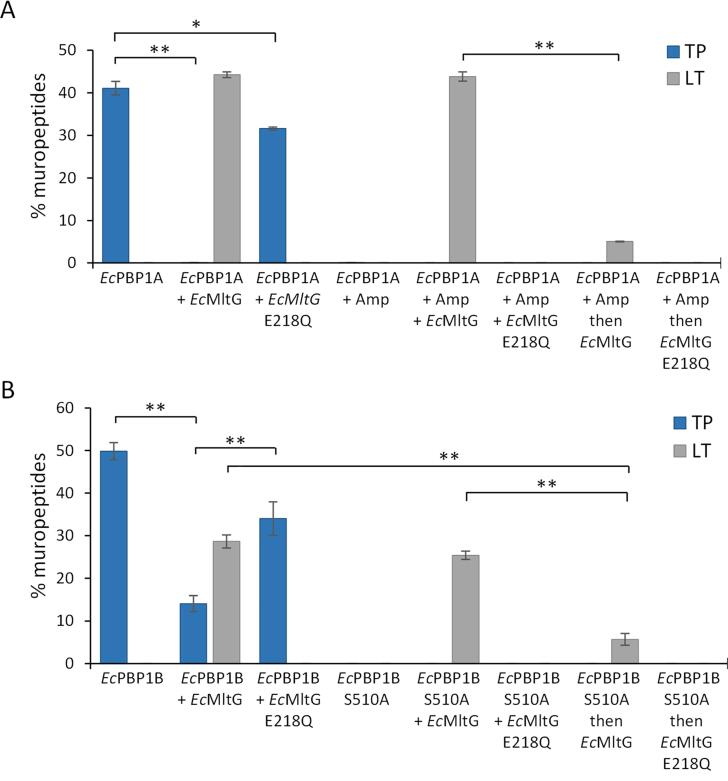
*Ec*MltG favours ongoing glycan strand polymerisation for LT activity. Levels of crosslinked muropeptides (TP: blue columns) and LT product (LT: grey columns) obtained after cellosyl-digestion of the PG synthesised by *Ec*PBP1A, *Ec*PBP1B and/or *Ec*MltG, from *E. coli*-type lipid II. Values are the mean ± variation of two independent experiments. *Ec*MltG has a lytic transglycosylase activity in the presence of *Ec*PBP1A or *Ec*PBP1B. *Ec*MltG reduces the TPase activity of *Ec*PBP1A and *Ec*PBP1B. *Ec*PBP1B S510A and *Bs*MltG E218Q, catalytically inactive versions (controls); Amp, Ampicillin. **, P < 0.01; *, P < 0.05 (T-test). (For interpretation of the references to colour in this figure legend, the reader is referred to the web version of this article.)

*Bs*MltG was more active in the presence of ongoing glycan strand synthesis by *Bs*PBP1, and reduced the TPase activity of the latter. Since *Ec*MltG showed weak LT activity against *E. coli* PG and interacts with *Ec*PBP1B, we hypothesised that *Ec*MltG is active during PG synthesis by *Ec*PBP1A or *Ec*PBP1B. To test this hypothesis, we performed PG synthesis experiments using *Ec*PBPs and *E. coli*-type lipid II. *Ec*PBP1A alone produced PG with 41.1% of the peptides present in crosslinks (Fig. 2 and S6), however, *Ec*PBP1A produced no crosslinks in the presence of *Ec*MltG and the sample contained 44.3% PentaAnh (Fig. 2 and S6), suggesting that *Ec*MltG inhibits the TPase activity of *Ec*PBP1A. *Ec*PBP1A produced fewer crosslinks (31.6%) also in the presence of catalytically inactive *Ec*MltG E218Q, as expected this sample did not contain PentaAnh, suggesting that inactive *Ec*MltG is also able to reduce the TPase activity of *Ec*PBP1A. Additionally, the sample prepared with *Ec*PBP1A (+Amp) and then digested with *Ec*MltG contained significantly lower LT activity (5.0%) (Fig. 2 and S6), suggesting that, similar to *Bs*MltG, *Ec*MltG is most active during ongoing PG synthesis.

*Ec*PBP1B alone produced PG with 49.9% peptides in crosslinks but in the presence of *Ec*MltG approximately 3.5 × fewer peptides were present in crosslinks (14.1%), and 28.7% PentaAnh was produced by *Ec*MltG (Fig. 2 and S7). The presence of *Ec*MltG E218Q resulted also in a significant decrease in TPase products generated by *Ec*PBP1B (34.0%). *Ec*MltG produced 25.4% PentaAnh in the presence of ongoing PG synthesis by a TPase inactive *Ec*PBP1B S510A, close to the levels observed in the presence of active *Ec*PBP1B with *Ec*MltG (Fig. 2 and S7). However, *Ec*MltG produced only 5.7% PentaAnh when it was added after PG synthesis by *Ec*PBP1B S510A, confirming that *Ec*MltG is more active during ongoing glycan strand polymerisation by either *Ec*PBP1A or *Ec*PBP1B.

### EcMltG has lower activity in the presence of activated EcPBP1A and EcPBP1B.

**Fig. 3:**
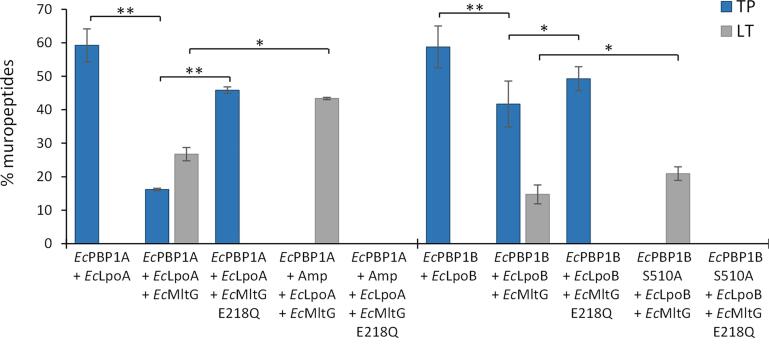
*Ec*MltG is active in the presence of activated *Ec*PBP1A and *Ec*PBP1B. Level of crosslinked muropeptides (TP: blue columns) and LT products (grey columns) obtained after cellosyl-digestion of PG synthesised by Lpo-activated *Ec*PBP1A and *Ec*PBP1B in the presence or absence of *Ec*MltG versions, in assays with *E. coli*-type lipid II. Values are the mean ± variation of two independent experiments. *Ec*MltG reduces the TPase activity of both, activated *Ec*PBP1A and *Ec*PBP1B. *Ec*MltG had lower LT activity in the presence of Lpo-activated *Ec*PBP1A or *Ec*PBP1B, compared to *Ec*PBP1A or *Ec*PBP1B alone. *Ec*PBP1B S510A and *Ec*MltG E218Q, catalytically inactive versions (controls); Amp, Ampicillin. **, P < 0.01; *, P < 0.05 (T-test). (For interpretation of the references to colour in this figure legend, the reader is referred to the web version of this article.)

The outer membrane-anchored lipoprotein LpoA activates *Ec*PBP1A by direct interaction and stimulates its TPase activity, whereas, LpoB interacts with *Ec*PBP1B and stimulates its GTase and TPase activities (Egan et al., 2014, Lupoli et al., 2014, Typas et al., 2010). To test the effect of MltG on glycan strand synthesis by Lpo-activated PBPs, we assayed the GTase and TPase activity of *Ec*PBPs, and the LT activity of *Ec*MltG, in the presence of the PBP activators. As expected, the activation of *Ec*PBP1A by LpoA resulted in increased levels of peptides present in crosslinks (59.2%) compared to *Ec*PBP1A alone (41.1%) (Fig. 2, Fig. 3). The addition of *Ec*MltG resulted in a significant decrease in peptides present in crosslinks to 16.2% (Fig. 3 and S8A) which, however, was above the 0% peptides in crosslinks without LpoA (Fig. 2). These results suggest that the activation of *Ec*PBP1A rescued some of the TPase activity of PBP1A despite the presence of *Ec*MltG. On the other hand, *Ec*MltG synthesised 26.7% PentaAnh in the presence of *Ec*PBP1A and LpoA (Fig. 3 and S8A), which was significantly lower than in the presence of *Ec*PBP1A without LpoA (44.3%) (Fig. 2). Blocking the TPase activity of *Ec*PBP1A with ampicillin (*Ec*PBP1A, Amp, LpoA, *Ec*MltG sample) resulted in a significant increase in PentaAnh (43.4%). These results show that the increase in TPase activity is matched by a decrease in LT activity, and vice versa, and suggest that *Ec*PBP1A and *Ec*MltG compete with each other for the glycan strand substrates.

*Ec*PBP1B produced PG with 58.8% peptides in crosslinks in the presence of LpoB (Fig. 3 and S8B), however, the addition of *Ec*MltG resulted in a significant decrease in TPase products to 41.7% and the production of 14.7% PentaAnh. The sample of *Ec*PBP1B with LpoB and *Ec*MltG E218Q also showed lower levels of TPase products (49.3%) compared to *Ec*PBP1B with LpoB (Fig. 3 and S8B), suggesting that catalytically active or inactive *Ec*MltG compromises the TPase activity of LpoB-activated *Ec*PBP1B. *Ec*MltG produced 20.9% PentaAnh in the presence of *Ec*PBP1B S510A and LpoB (Fig. 3) compared to 25.4% in the presence of *Ec*PBP1B S510A alone (Fig. 2B), suggesting that increased glycan synthesis rate resulted in a small decrease in *Ec*MltG LT activity. Taken together, these results suggest that *Ec*MltG processes growing PG strands and this LT activity decreases the TPase activity of class A PBPs, presumably by competing for the same PG strands, despite the activation by the Lpo protein.

### MltG has no effect on the GTase activity of class A PBPs

The effect of MltG on the GTase activity of PBPs was tested using fluorescent-labelled Dansyl-lipid II. The polymerisation of glycan strands from Dansyl-lipid II and the digestion of these by a muramidase results in a decrease in fluorescence over time. As predicted, *Ec*MltG or *Bs*MltG alone did not cause a decrease in fluorescence over time demonstrating that both enzymes have no GTase activity (Fig. S9A and B). *Bs*PBP1 alone, or *Bs*PBP1 with *Bs*MltG showed similar decrease in fluorescence showing that *Bs*MltG does not affect the GTase activity of *Bs*PBP1 (Fig. S9A). Similarly, *Ec*PBP1A showed a decrease in fluorescence alone or in the presence of *Ec*MltG and/or LpoA, suggesting that neither *Ec*MltG nor LpoA affects the GTase activity of *Ec*PBP1A (Fig. S9B). Confirming previous data (Egan et al., 2014), the addition of LpoB resulted in a faster decrease in fluorescence by *Ec*PBP1B, but *Ec*MltG had no effect on *Ec*PBP1B alone or *Ec*PBP1B/LpoB (Fig. S9C). These data show that *Ec*MltG has no effect on the GTase activity of PBP1B, or its activation by LpoB.

### EcMltG and BsMltG process glycan strands after the seventh disaccharide repeat

**Fig. 4:**
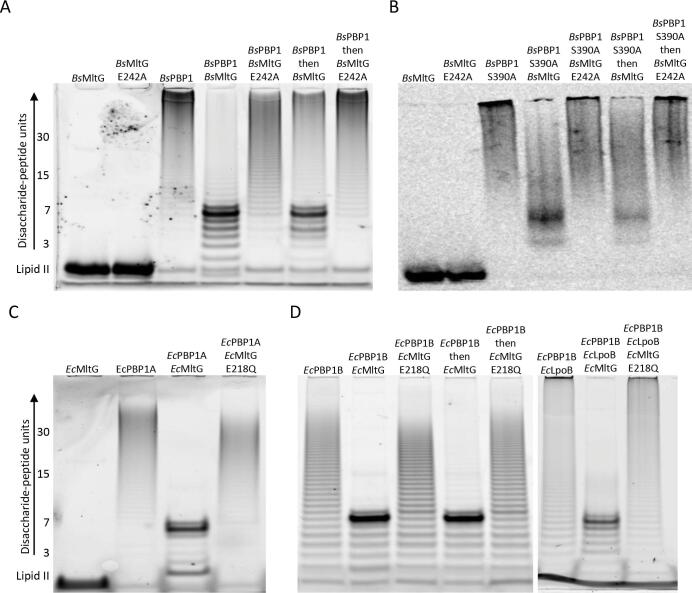
*Bs*MltG and *Ec*MltG cleave PG glycan strands after the seventh disaccharide unit. Glycan strand length assays for products of *Bs*PBP1, *Ec*PBP1A and *Ec*PBP1B formed in the presence of MltG from *B. subtilis* or *E. coli*. *B. subtilis* Proteins were incubated with fluorescent ATTO-550 lipid II (A) or [^14^C]-GlcNAc-labelled lipid II (B), and ampicillin to block cross-linking. (C) *E. coli* proteins incubated with fluorescent lipid and ampicillin. PG synthesised by the enzymes was resolved by SDS-PAGE and visualised by fluorescence imaging. The position of the substrates is marked by ‘Lipid II’, the length of the glycan strand is given in disaccharide units.

*Ec*MltG and *Bs*MltG cleaved newly synthesised glycan strands, suggesting a role in glycan length determination. To test this hypothesis, the activity of PBPs and MltG was tested against radioactive or fluorescent (ATTO-550) lipid II in the presence of ampicillin, and the resulting glycan strands were analysed by SDS-PAGE. *Bs*PBP1 alone produced long glycan strands that migrated at the top of the gel (Fig. 4A). The presence of *Bs*MltG and not *Bs*MltG E242A caused the formation of shorter glycan strands, with a distinctive increase in strands with 7 disaccharide units. Adding *Bs*MltG after the strands were synthesized, also generated glycan strands with 7 disaccharide units, however, longer glycan strands were also detected suggesting that *Bs*MltG was less active, consistent with the previous experiments (Fig. 1B). Testing the activity of *Bs*MltG and/or *Bs*PBP1 against radioactive lipid II without the fluorophore produced similar results with a significant increase in short strands, albeit the glycan strand separation in the gel was poorer in this case (Fig. 4B). *Ec*MltG was also tested using the fluorescent lipid II in the presence of *Ec*PBP1A and *Ec*PBP1B (Fig. 4C and D). *Ec*PBP1A or *Ec*PBP1B alone produced long glycan strands, however, the presence of *Ec*MltG decreased the glycan strand length and, again, there was a significant increase in strands with 7 disaccharide units (Fig. 4C–D). This suggests that MltG from both species produces glycan strands with 7 disaccharide units when it cleaves the nascent strands during GTase reactions. Next, we tested if a faster rate of glycan synthesis would affect PG cleavage by *Ec*MltG by testing *Ec*PBP1B with LpoB and *Ec*MltG against lipid II (Fig. 4D). *Ec*PBP1B and LpoB produced glycan strands with a broader length distribution (Egan et al., 2018). The addition of *Ec*MltG resulted in a similar processing of the glycan strands and a clear increase in the strands with 7 disaccharide repeats, suggesting that the length of strands generated by *Ec*MltG is independent from the rate of glycan polymerisation (Fig. 4D).

## Discussion

**Fig. 5:**
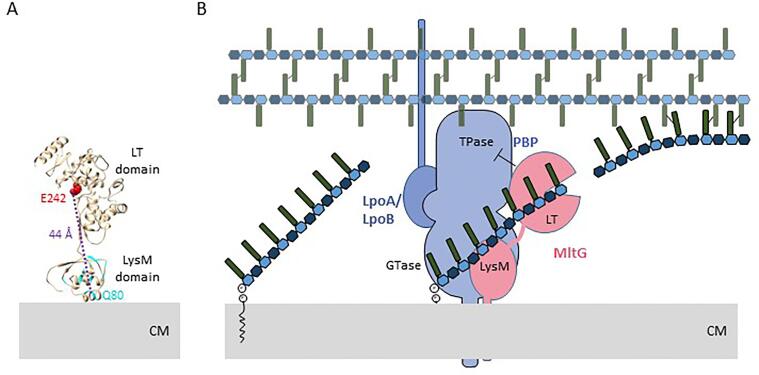
Model of glycan strand cleavage by MltG. (A) *Bs*MltG residues were modelled using the crystal structure of Lmo1499 (PDB: 4IIW) as template (Yang et al., 2020). Highly conserved residues in the LysM domain are in cyan (Fig. S1), and the catalytic E242 residue is in red. The distance from the LT catalytic residue E242 to one of the distant and conserved LysM domain residues, Q80, is ~ 44 Å. (B) MltG is recruited to PG synthesis sites, presumably by interacting with PBPs, and competes with their TPase activity, cleaving in the nascent glycan strands to produce strands with 7 disaccharide units. (For interpretation of the references to colour in this figure legend, the reader is referred to the web version of this article.)

*Bs*MltG showed 67% sequence similarity and 53% identity to Lmo1499, a membrane bound lytic transglycosylase from *Listeria monocytogenes* (*Lm*MltG). *Lm*MltG has a transmembrane region followed by a LysM domain, involved in PG binding (Buist et al., 2008), and a catalytic domain close to the C-terminus of the protein (PDB: 4IIW), and this domain organization is conserved in *Bs*MltG (Fig. 5A and S1). The LysM domain of MltG could mediate the binding to newly synthesised glycan strand in close proximity to the cytoplasmic membrane (Fig. 5). Interestingly, MltG produced glycan strands with 7 disaccharide residues, in the presence or absence of ongoing PG synthesis, suggesting that MltG binds a nascent glycan strand protruding from the membrane and cleaves it after 7 disaccharide units. In the model of *Bs*MltG the distance between the LT catalytic residue E242 and one of the distant and conserved LysM domain residues Q80 is ~ 44 Å (Fig. 5A and S1), which corresponds to the length of 4–5 disaccharide units, given the length of one disaccharide unit of ~ 10 Å (Fig. 5A) (Carlström, 1962, Carlström, 1957). Presumably, the length of the glycan strand produced by MltG depends also on the distance of the LT active site to the terminal disaccharide unit. Future work will explore whether MltG can be used to produce a sufficient amount of glycan strands with a desired length for biochemical or structural studies.

Maintaining the integrity of the PG layer is crucial to protect the cell from bursting due to its turgor. In this manuscript, we showed that MltG cleaves in nascent PG strands produced by class A PBPs and competes with their TPase activity even in the presence of the Lpo activators. These results are consistent with published data showing that the loss of MltG function suppressed a lethal deficiency in class A PBP activity (Bohrhunter et al., 2020), presumably by alleviating the reduction of TPase activity exerted by MltG on PBPs, and the decrease in PG cleavage. Interestingly, the absence or overexpression of *Ec*MltG has no effect on the fitness of wild type *E. coli* cells (Yunck et al., 2016).

What is the cellular function of MltG? It was previously hypothesized that MltG is a 'terminase' for glycan strand polymerization (Yunck et al., 2016). Prior to Yunck et al., 2016, the term 'terminase' was only used for an enzyme involved in bacteriophage lambda genome packaging (Catalano, 2000) and not for enzymes cleaving in nascent polymers. MltG could generate short glycan strands either by terminating the GTase activity of the class A PBP after the nascent glycan strand reaches a certain length, causing glycan strand release. Alternatively, MltG cleaves within the nascent glycan strand during its synthesis, without affecting the GTase reaction. Our data support the latter hypothesis since the addition of MltG did not show an effect on the GTase activity of class A PBPs, which continued in the presence of MltG (Fig. S9). Additionally, MltG might be needed to detach a nascent glycan strand from the membrane after it has been incorporated into the PG layer (Yunck et al., 2016; Fig. 5B). Indeed, pulse chase experiments showed the formation of MurNAcAnh-containing LT products shortly after glycan strand polymerization suggesting that LT enzymes may act in close proximity to PG synthases or as part of the PG synthesis machinery (Burman and Park, 1983, Glauner and Höltje, 1990). Consistent with this hypothesis, *Bs*MltG interacts with *Bs*PBP1 and *Bs*PBP2B, and *Ec*MltG associates with GTase active *Ec*PBP1B and RodA (Bohrhunter et al., 2020, Yunck et al., 2016), which presumably targets MltG to the newly synthesised PG strands (Fig. 5B). However, this function does not appear to be essential and it remained unclear whether the detachment of glycan strands from the membrane occurs in the *mltG* mutant and, if so, if it is important. Our discoveries that MltG produces 7-disaccharide long glycan strands, which was corroborated by work done in the group of Suzanne Walker (preprint on BioRxiv, Taguchi and Walker, 2021) and that it competes with the TPase function of class A PBPs over the use of glycan strands, could point to another function. We hypothesize that MltG utilizes some of the nascent PG made by PG synthases to produce short glycan strands that are used for a yet unknown process. These glycan strands could be used to initiate the polymerisation of a new glycan strand, or they could be used to repair defects in the PG layer. For example, the GTase function of PBP1B is needed together with the LD-transpeptidase LtdD and the DD-carboxypeptidase PBP6A for cell survival upon the inhibition of LPS export to the outer membrane (Morè et al., 2019). It remains to be seen whether MltG participates in the repair of defective PG or has other cellular functions.

## Conclusion

Our results suggest that inner-membrane bound lytic transglycosylases like MltG can modulate PG synthesis not only through their catalytic activities but also by interacting with and regulating the activities of PG synthases, and ultimately determining the structure of the PG.

## CRediT authorship contribution statement

**Jad Sassine:** Conceptualization, Validation, Formal analysis, Investigation, Writing - original draft, Writing - review & editing, Visualization. **Manuel Pazos:** Investigation, Writing - review & editing. **Eefjan Breukink:** Resources, Writing - review & editing. **Waldemar Vollmer:** Conceptualization, Methodology, Validation, Data curation, Writing - original draft, Writing - review & editing, Supervision, Project administration, Funding acquisition.

## Declaration of Competing Interest

The authors declare that they have no known competing financial interests or personal relationships that could have appeared to influence the work reported in this paper.
