# Supplementary material for: Lytic transglycosylase MltG cleaves in nascent peptidoglycan and produces short glycan strands
Source: Cell Surf. 2021 May 1;7:100053. doi: 10.1016/j.tcsw.2021.100053 (PMC8135044; doi:10.1016/j.tcsw.2021.100053)
Supplement: Supplementary data 1 [file mmc1.pdf]

SUPPLEMENTARY DATA

Lytic transglycosylase MltG cleaves in nascent peptidoglycan and produces short glycan strands

Jad Sassine[1‡], Manuel Pazos[1], Eefjan Breukink[2], and Waldemar Vollmer[1*].

[1]Centre for Bacterial Cell Biology, Biosciences Institute, Faculty of Medical Sciences, Newcastle University, Newcastle upon Tyne, UK.

[2]Membrane Biochemistry and Biophysics, Bijvoet Centre of Biomolecular Research, Department of Chemistry, Faculty of Science, Utrecht University, Utrecht, Netherlands.

[‡] Current address: Department of Biochemistry, University of Oxford, Oxford, UK.

* To whom correspondence may be addressed. E-mail: Waldemar.vollmer@ncl.ac.uk

Index

Supplementary figures

- Fig. S1. Amino acid sequence alignment of *Bs*SleB*, Bs*MltG, *Ec*MltG and *Lm*MltG.
- Fig. S2. *Bs*MltG interacts with *Bs*PBP2B.
- Fig. S3. RP-HPLC analysis of muropeptides produced from *B. subtilis* PG digest with *Bs*MltG and/or cellosyl.
- Fig. S4. HPLC chromatograms corresponding to data shown in Figure 1B.
- Fig. S5. Lytic transglycosylase activity of *Bs*MltG.
- Fig. S6. HPLC chromatograms corresponding to data shown in Figure 2A.
- Fig. S7. HPLC chromatograms corresponding to data shown in Figure 2B.
- Fig. S8. HPLC chromatograms corresponding to data shown in Figure 3.
- Fig. S9. MltG has no effect on the GTase activity of PBPs.

Supplementary tables

- Table S1. List of strains.
- Table S2. List of plasmids.
- Table S3. List of primers.

References

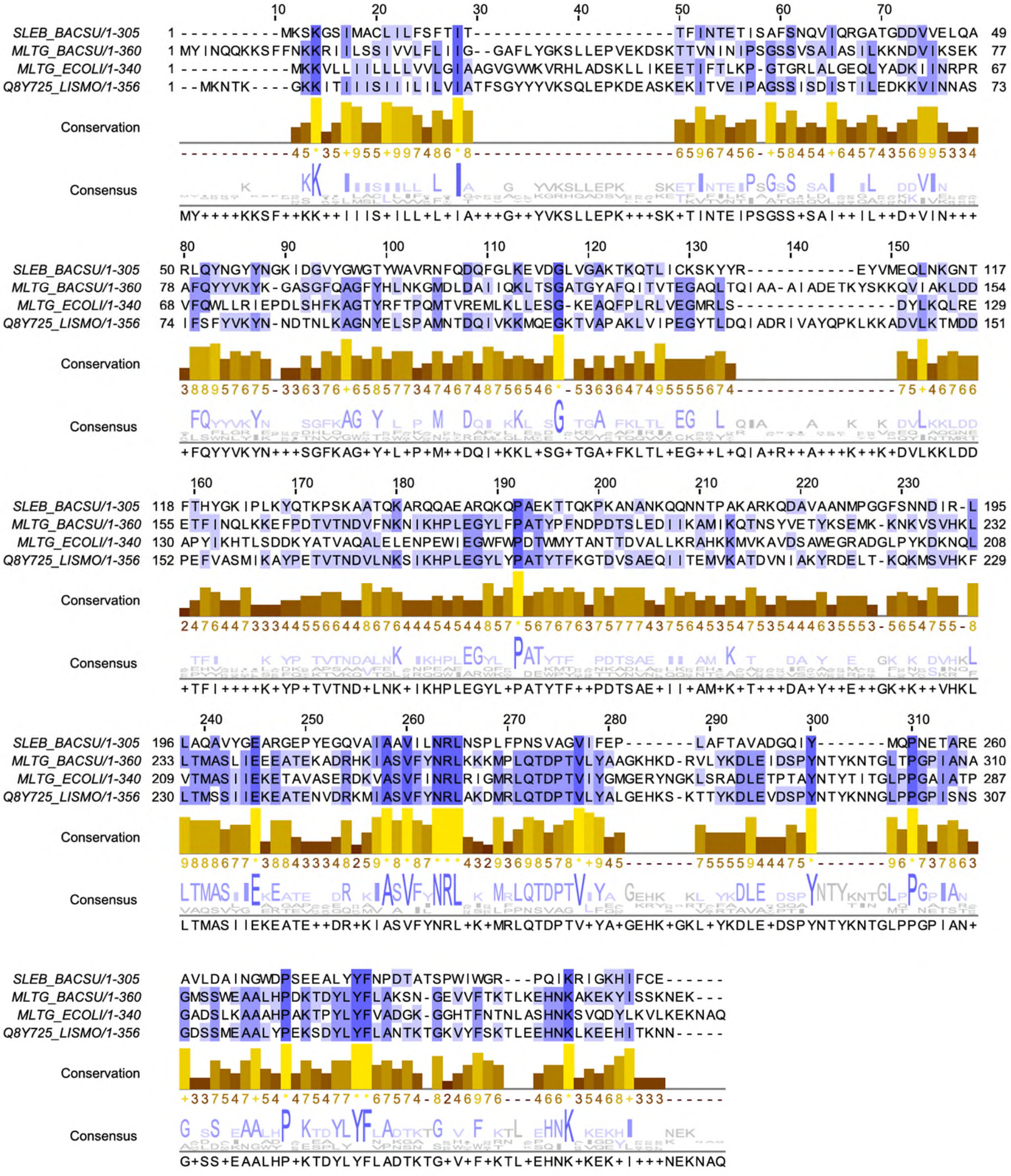

Figure S1. Amino acid sequence alignment for *Bs*SleB, *Bs*MltG, *Ec*MltG and *Lm*MltG. The LysM domain is predicted to range from residues 50 to 135, and LT domain from 150 to 340. The active site glutamate residue E218 in *Ec*MltGis conserved across species (Yunck *et al.*, 2016).

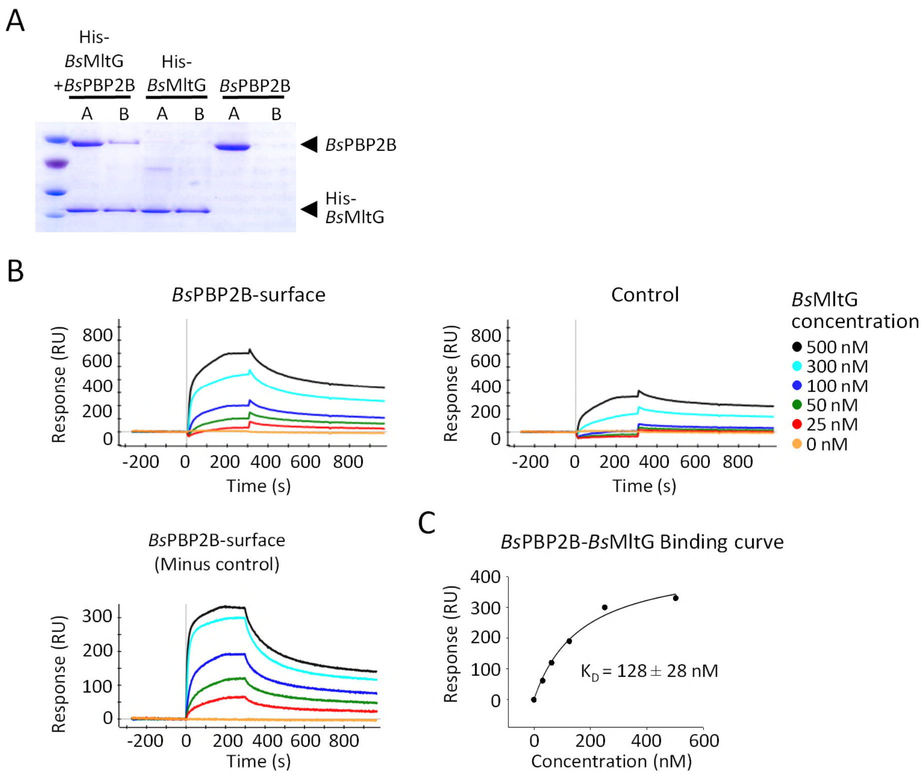

Figure S2. *Bs*MltG interacts with *Bs*PBP2B.
(**A**) Pull-down assays performed to test if *Bs*MltG interacts with *Bs*PBP2B. Coomassie stained SDS-PAGE analysis showing that His-*Bs*MltG and *Bs*PBP2B were detected in both the applied and the bound fractions suggesting that His-*Bs*MltG pull-down *Bs*PBP2B. A, applied fraction; B, bound fractions. (**B**) SPR sensorgrams showing the response for *Bs*MltG when injected over a surface with immobilized *Bs*PBP2B or a control surface, plotted against time. The signal for the *Bs*PBP2B-surface was higher than for the control surface upon *Bs*MltG injection. (**C**) The response values during equilibrium were plotted against injected *Bs*MltG concentrations. The K_D of the *Bs*PBP2B-*Bs*MltG interaction was determined by non-linear regression using Sigma Plot software and given as mean ± standard deviation of three independent experiments.

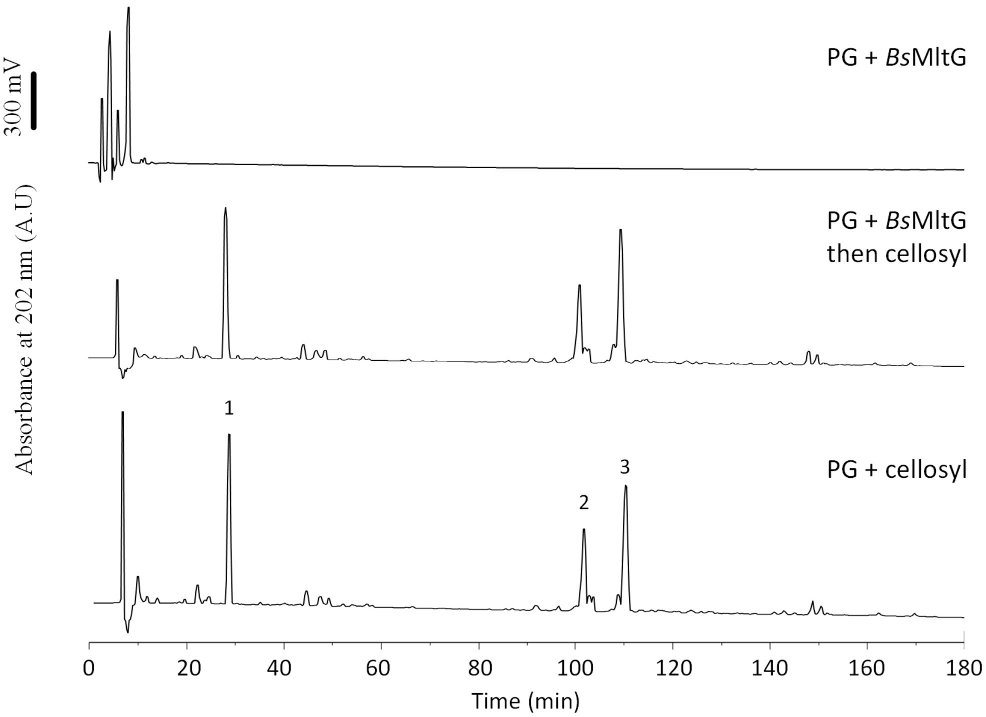

Figure S3. RP-HPLC analysis of muropeptides produced from *B. subtilis* PG digest with *Bs*MltG and/or cellosyl.

PG from *B. subtills* was incubated with *Bs*MltG and the sample was reduced with sodium borohydride and analysed by HPLC (Top chromatogram). As a control sample, the PG was incubated with *Bs*MltG and then digested with cellosyl, followed by reduction and HPLC analysis (Middle chromatogram). As another control sample, *B. subtills* PG was digested with cellosyl followed by reduction and HPLC analysis. Elution chromatograms were similar to previously published chromatograms of muropeptides generated from wild type *B. subtills* PG digests (Atrih *et al.*, 1999; Sassine *et al.*, 2020). PG digested with *Bs*MltG did not release muropeptides, on the contrary to PG digest by *Bs*MltG and cellosyl, suggesting that *Bs*MltG is inactive against PG. Peaks eluting between 0 and 10 min correspond to buffers components. The major eluted peaks correspond to: 1, Tri(NH_2), GlcNAc-MurNAc(r)-L-Ala-D-Glu-meso-diaminopimelic acid (amidated); 2, TetraTri(NH_2), bis-disaccharide tetratripeptide with amidation of one of the m-Dap residues; 3, TetraTri(NH_2)$_2$, bis-disaccharide tetratripeptide with amidation of both m-Dap residues.

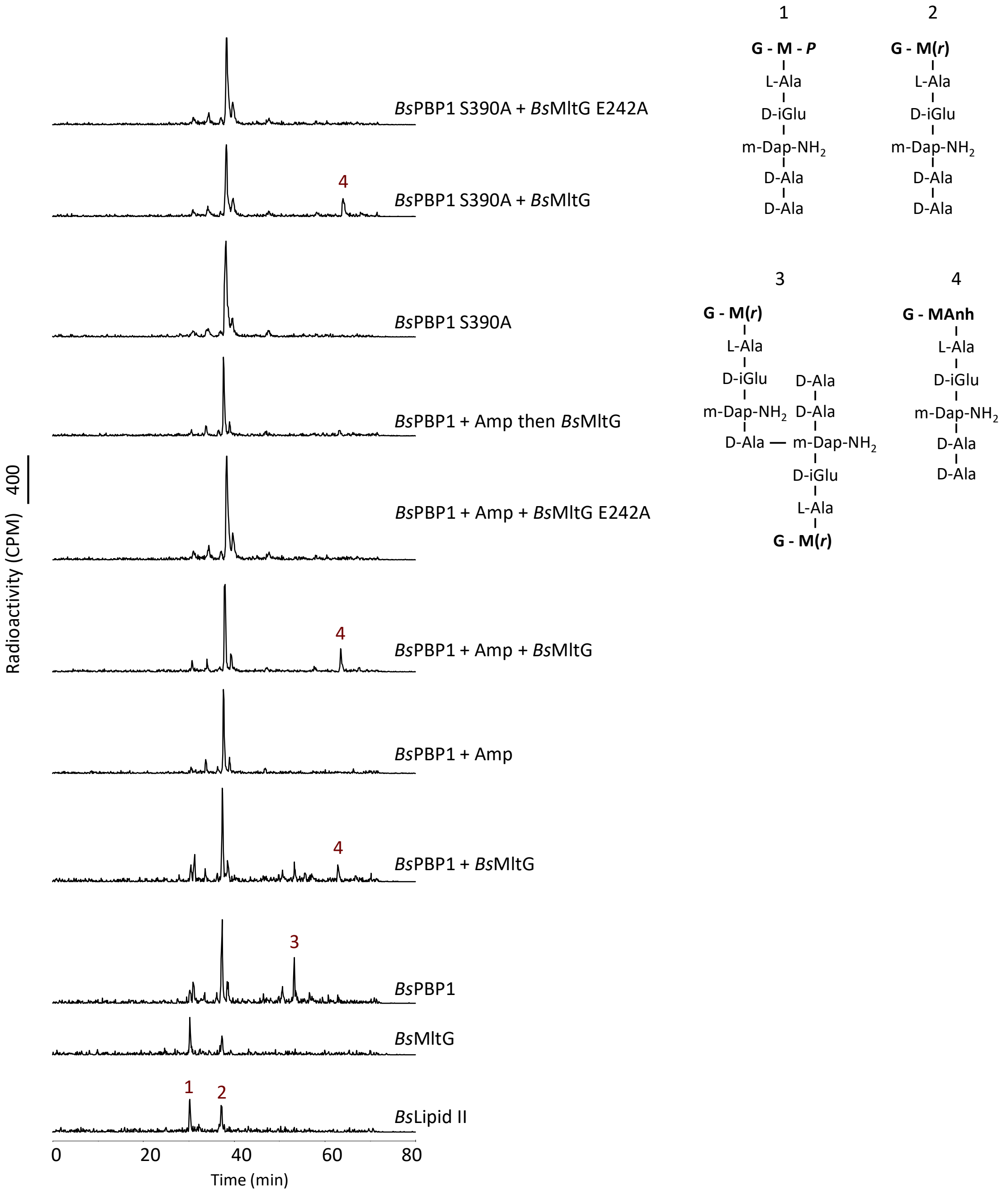

Figure S4. HPLC chromatograms corresponding to data shown in Figure 1B.

HPLC analysis of new peptidoglycan synthesised by *Bs*PBP1 or *Bs*PBP1 S390A in the presence or absence of ampicillin and *Bs*MltG or *Bs*MltG E242A. The PG was digested with cellosyl, reduced with sodium borohydride and separated by HPLC. Muropeptide structures are shown next to the chromatograms. Muropeptide 3 is a TP product, muropeptide 4 is an LT product. G, *N*-acetylglucosamine; M(r), *N*-acetylmuramitol; *P*, phosphate; MAnh, 1,6-anhydro-*N*-acetylmuramic acid; L-Ala, L-alanine; D-Ala, D-alanine; D-iGlu, D-iso-glutamate; m-Dap, meso-2,6-diaminopimelic acid; NH_2, amido group at m-Dap (amidation).

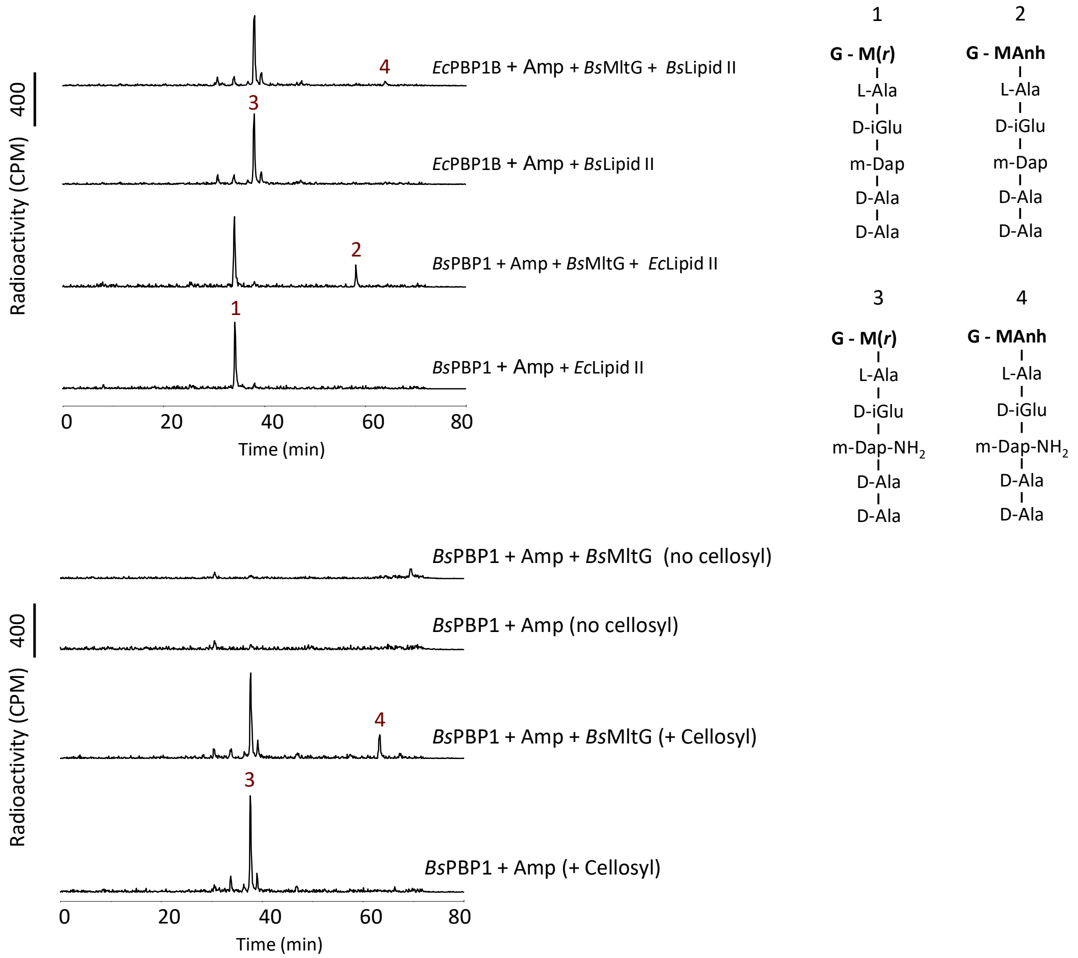

Figure S5. Lytic transglycosylase activity of *Bs*MltG.

HPLC analysis of new peptidoglycan synthesised by *Bs*PBP1 or *Ec*PBP1B using *E. coli* or *B. subtilis* lipid II in the presence or absence of ampicillin, *Bs*MltG and *Bs*MltG E242A. Muropeptide structures are shown next to the chromatograms. Muropeptides 2 and 4 are LT products. *Bs*MltG showed a significantly higher activity when tested in the presence of *Bs*PBP1 compared to *Ec*PBP1B, suggesting that *Bs*MltG favours ongoing PG synthesis by *Bs*PBP1 for activity. *Bs*MltG products are detectable only after further cleaving the glycan material by cellosyl showing that *Bs*MltG is an endo-enzyme cleaving glycosidic bonds within the glycan strands. G, *N*-acetylglucosamine; M(r), *N*-acetylmuramitol; MAnh, 1,6-anhydro-*N*-acetylmuramic acid; L-Ala, L-alanine; D-Ala, D-alanine; D-iGlu, D-iso-glutamate; m-Dap, meso-2,6-diaminopimelic acid; NH_2, amido group.

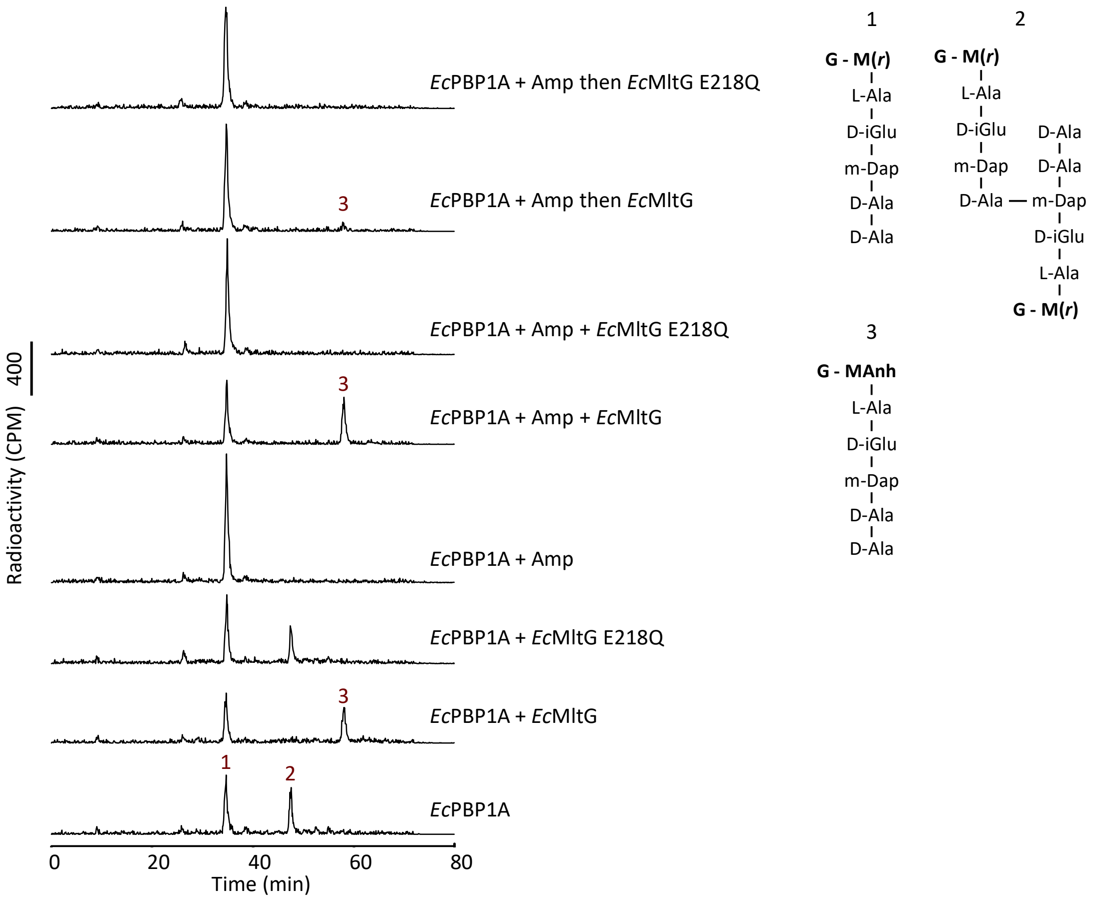

Figure S6. HPLC chromatograms corresponding to data shown in Figure 2A.
HPLC analysis of new peptidoglycan synthesised by *Ec*PBP1A in the presence or absence of ampicillin and *Ec*MltG or *Ec*MltG E218Q. The PG was digested with cellosyl, reduced with sodium borohydride and separated by HPLC. Muropeptide structures are shown next to the chromatograms. Muropeptide 2 is a TP product, muropeptide 3 is an LT product. G, *N*-acetylglucosamine; M(r), *N*-acetylmuramitol; MAnh, 1,6-anhydro-*N*-acetylmuramic acid; L-Ala, L-alanine; D-Ala, D-alanine; D-iGlu, D-iso-glutamate; m-Dap, meso-2,6-diaminopimelic acid.

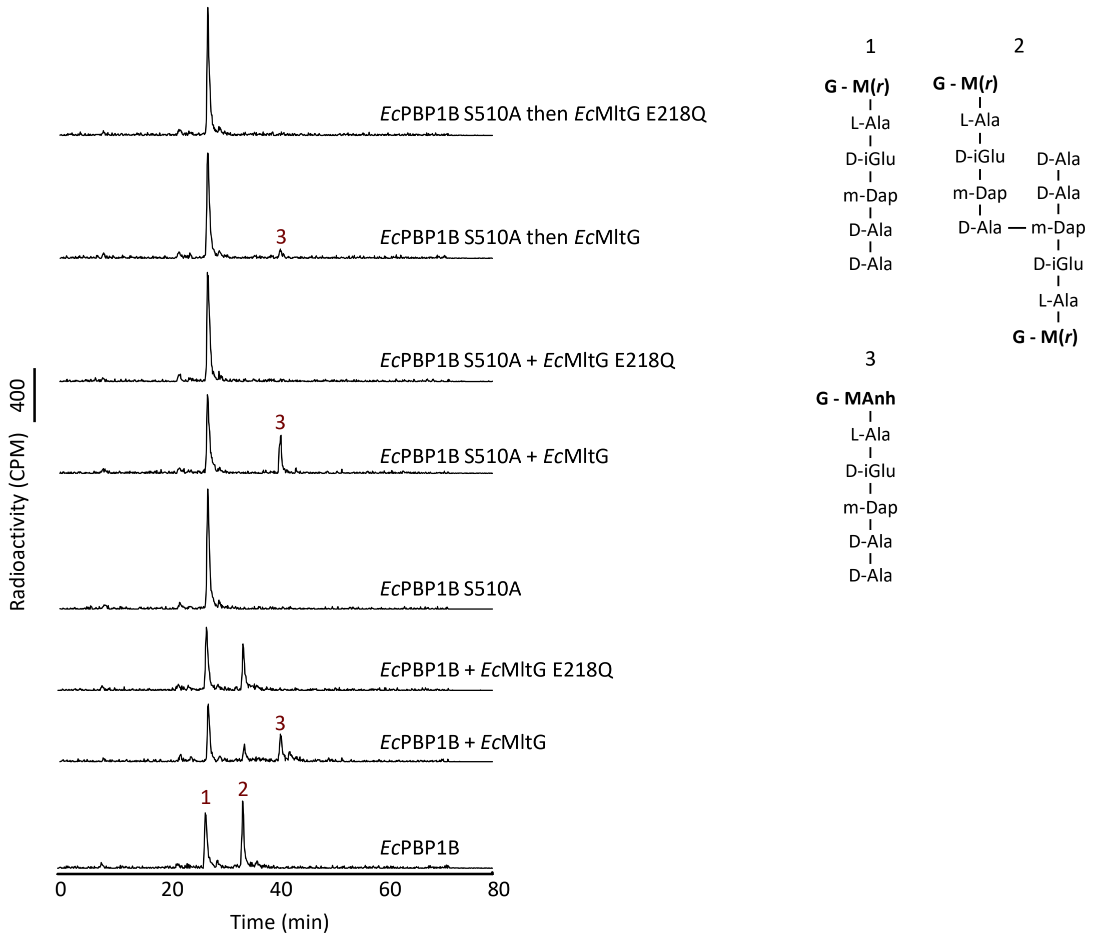

Figure S7. HPLC chromatograms corresponding to data shown in Figure 2B.
HPLC analysis of new peptidoglycan synthesised by *Ec*PBP1B or *Ec*PBP1B S510A in the presence or absence of ampicillin and *Ec*MltG or *Ec*MltG E218Q. The PG was digested with cellosyl, reduced with sodium borohydride and separated by HPLC. Muropeptide structures are shown next to the chromatograms. Muropeptide 2 is a TP product, muropeptide 3 is an LT product. G, *N*-acetylglucosamine; G, *N*-acetylglucosamine; M(r), *N*-acetylmuramitol; MAnh, 1,6-anhydro-*N*-acetylmuramic acid; L-Ala, L-alanine; D-Ala, D-alanine; D-iGlu, D-iso-glutamate; m-Dap, meso-2,6-diaminopimelic acid.

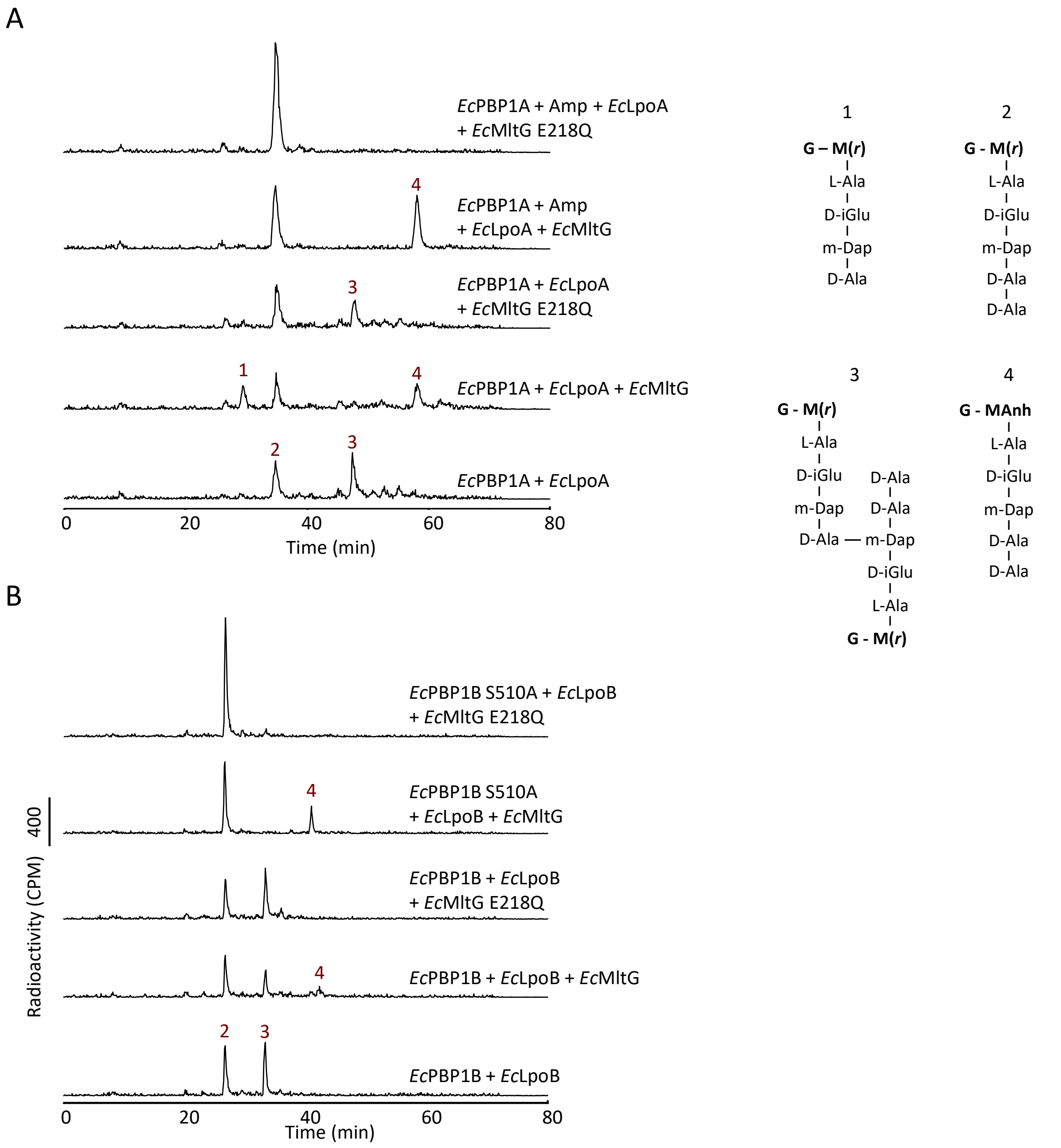

Figure S8. HPLC chromatograms corresponding to data shown in Figure 3.

HPLC analysis of new peptidoglycan synthesised by *Ec*PBP1A, *Ec*PBP1B and *Ec*PBP1B S510A in the presence or absence of ampicillin and LpoA, LpoB, *Ec*MltG or *Ec*MltG E218Q. The PG was digested with cellosyl, reduced with sodium borohydride and separated by HPLC. *Ec*MltG produced PentaAnh in the presence of (A) *Ec*PBP1B and LpoB, and (B) *Ec*PBP1A and LpoA. Muropeptide structures are shown next to the chromatograms. Muropeptide 3 is a TP product, muropeptide 4 is an LT product. G, *N*-acetylglucosamine; M(r), *N*-acetylmuramitol; MAnh, 1,6-anhydro-*N*-acetylmuramic acid; L-Ala, L-alanine; D-Ala, D-alanine; D-iGlu, D-iso-glutamate; m-Dap, meso-2,6-diaminopimelic acid.

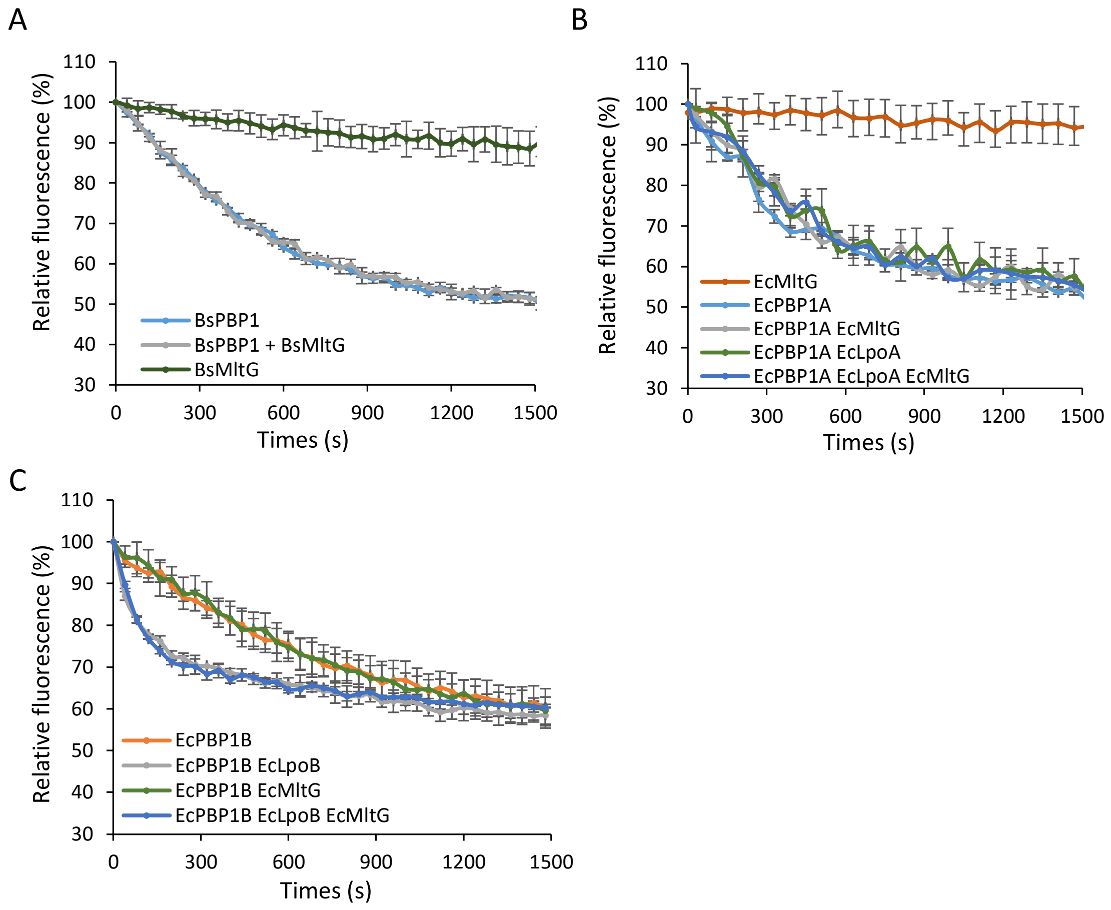

Figure S9. MltG has no effect on the GTase activity of PBPs.

Plots represent the GTase activity of *Bs*PBP1, *Ec*PBP1B or *Ec*PBP1A in the presence of LpoA, LpoB, *Bs*MltG and/or *Ec*MltG, using fluorescent dansyl-lipid II as substrate. GTase activity causes a decrease in the fluorescence signal over time. *Bs*MltG or *Ec*MltG were inactive against dansyl-lipid II. *Bs*MltG had no effect on the GTase activity of *Bs*PBP1. *Ec*MltG had no effect on the GTase activity of *Ec*PBP1A or *Ec*PBP1B. *Ec*MltG had no effect on the activation of *Ec*PBP1B by LpoB. Values represent the mean ± standard deviation of three independent experiments.

Table S1. List of strains.

Strain	Genotype	Source/Comment
DH5α	*E. coli* F^{-} φ*80lacZ*Δ*M15*, Δ(*lacZYArgF*)*U196*, *recA1*, *endA1*, *hsdR17*, (r_{k-}, m_{k+}), *phoA*, *supE44*, λ^{-}, $thi^{-}1$, *gyrA96*, *relA1*	Invitrogen
BL21(DE3)	*E. coli* B F^{-} *ompT gal dcm lon* $hsdS_B(r_B^{-}m_B^{-})$ λ(DE3 [*lacI lacUV5-T7p07 ind1 sam7 nin5*]) $[malB^{+}]_{K\text{-}12}(\lambda^S)$	Novagen
B. subtilis 168CA	*trpC2*	Laboratory collection
E. coli BW25113	Wild type strain	(Datsenko and Wanner, 2000)

Table S2. List of plasmids.

Plasmid	Characteristics	Reference/Source
pET-28a(+)	*kan* P_{T7} *lacI*	Novagen
pET28-28a(+)::*ponA*	*kan* P_{T7} *ponA lacI*	(Cleverley et al., 2016; Rismondo et al., 2016)
pJS07	*kan* P_{T7} *pbpB lacI*	This work
pJS11	*kan* P*T7 ponA*(S390A) *lacI*	This work
pJS12	*kan* P_{T7} *yrrL lacI*	This work
pJS136	*kan* P_{T7} *yrrL E242A lacI*	This work
pJS154	*kan* P_{T7} *mltG lacI*	This work
pJS158	*kan* P_{T7} *mltG E218Q lacI*	This work
pDML924	*kan* P_{T7} *mrcB lacI*	(Terrak et al., 1999)
pDML924(S510A)	*kan* P_{T7} *mrcB S510A lacI*	(Leclercq et al., 2017)
pLpoB^{sol}	*kan* P_{T7} *lpoB lacI*	(Typas et al., 2010)
pTK1Ahis	*kan* P_{T7} *mrcA lacI*	(Born et al., 2006)
pET28-HisLpoA(sol)	*kan* P_{T7} *lpoA lacI*	(Typas et al., 2010)

Table S3. List of primers.

Name	5’-3’ oligonucleotide sequence	Reference/comment
JS128	ATGGCTAGCATGACTGGTGGAC	To PCR amplify pET-28a(+)
JS129	ATGGCTGCCGCGCGGCACCAG	To PCR amplify pET-28a(+)
JS134	CTGGTGCCGCGCGGCAGCCATATGTATATCAATCAGCAAAAAAAATCG	5’ *yrrL* (construction of pJS12)
JS135	TGTCCACCAGTCATGCTAGCCATGCGGAAAGCGAACAAAAGGAGAG	3’ *yrrL* (construction of pJS12)
JS155	GTCGGCTTTTTCAGTCGCTGCTTCTTCTATCAAAGAAGC	To construct pJS136 by side-directed mutagenesis
JS156	GCTTCTTTGATAGAAGAAGCAGCGACTGAAAAAGCCGAC	To construct pJS136 by side-directed mutagenesis
JS190	CTGGTGCCGCGCGGCAGCCATATGAAAAAAGTGTTATTGATAATCT	5’ *mltG* (construction of pJS154)
JS191	TGTCCACCAGTCATGCTAGCCATCTCAATGACGATATACTTACTG	3’ *mltG* (construction of pJS154)
JS193	GACGATGGCATCAATTATCGAAAAACAGACCGCCGTTGCCA	To construct pJS158 by side-directed mutagenesis
JS194	TGGCAACGGCGGTCTGTTTTTCGATAATTGATGCCATCGTC	To construct pJS158 by side-directed mutagenesis

References

Atrih, A., Bacher, G., Williamson, M.P., Foster, S.J., 1999. Analysis of peptidoglycan structure from vegetative cells of *Bacillus subtilis* 168 and role of PBP 5 in peptidoglycan maturation. J. Bacteriol. 181, 3956–3966. https://jb.asm.org/content/181/13/3956

Born, P., Breukink, E., Vollmer, W., 2006. In vitro Ssynthesis of Ccross-linked murein and its attachment to sacculi by PBP1A from *Escherichia coli*. J. Biol. Chem. 281, 26985–26993. https://doi.org/10.1074/jbc.M604083200

Cleverley, R.M., Rismondo, J., Lockhart-Cairns, M.P., Van Bentum, P.T., Egan, A.J.F., Vollmer, W., Halbedel, S., Baldock, C., Breukink, E., Lewis, R.J., 2016. Subunit arrangement in GpsB, a regulator of cell wall biosynthesis. Microb. Drug Resist. 22, 446–460. https://doi.org/10.1089/mdr.2016.0050

Datsenko, K.A., Wanner, B.L., 2000. One-step inactivation of chromosomal genes in *Escherichia coli* K-12 using PCR products. Proc. Natl. Acad. Sci. 97, 6640–6645. https://doi.org/10.1073/pnas.120163297

Leclercq, S., Derouaux, A., Olatunji, S., Fraipont, C., Egan, A.J.F., Vollmer, W., Breukink, E., Terrak, M., 2017. Interplay between Penicillin-binding proteins and SEDS proteins promotes bacterial cell wall synthesis. Sci. Rep. 7, 43306. https://doi.org/10.1038/srep43306

Rismondo, J., Cleverley, R.M., Lane, H. V, Großhennig, S., Steglich, A., Möller, L., Mannala, G.K., Hain, T., Lewis, R.J., Halbedel, S., 2016. Structure of the bacterial cell division determinant GpsB and its interaction with penicillin-binding proteins. Mol. Microbiol. 99, 978–998. https://doi.org/10.1111/mmi.13279

Sassine, J., Sousa, J., Lalk, M., Daniel, R.A., Vollmer, W., 2020. Cell morphology maintenance in *Bacillus subtilis* through balanced peptidoglycan synthesis and hydrolysis. Sci. Rep. 10, 17910. https://doi.org/10.1038/s41598-020-74609-5

Terrak, M., Ghosh, T.K., Van Heijenoort, J., Van Beeumen, J., Lampilas, M., Aszodi, J., Ayala, J.A., Ghuysen, J.M., Nguyen-Distèche, M., 1999. The catalytic, glycosyl transferase and acyl transferase modules of the cell wall peptidoglycan-polymerizing penicillin-binding protein 1b of *Escherichia coli*. Mol. Microbiol. 34, 350–364. https://doi.org/10.1046/j.1365-2958.1999.01612.x

Typas, A., Banzhaf, M., Van Den Berg Van Saparoea, B., Verheul, J., Biboy, J., Nichols, R.J., Zietek, M., Beilharz, K., Kannenberg, K., Von Rechenberg, M., Breukink, E., Den Blaauwen, T., Gross, C.A., Vollmer, W., 2010. Regulation of peptidoglycan synthesis by outer-membrane proteins. Cell. 143:1097–109. https://doi.org/10.1016/j.cell.2010.11.038

Yunck, R., Cho, H., Bernhardt, T.G., 2016. Identification of MltG as a potential terminase for peptidoglycan polymerization in bacteria. Mol. Microbiol. 99, 700–718. https://doi.org/10.1111/mmi.13258
